# Endothelial cell dysfunction in cardiac disease: driver or consequence?

**DOI:** 10.3389/fcell.2023.1278166

**Published:** 2023-10-25

**Authors:** Jules D. Allbritton-King, Guillermo García-Cardeña

**Affiliations:** ^1^ Department of Pathology, Center for Excellence in Vascular Biology, Brigham and Women’s Hospital and Harvard Medical School, Boston, MA, United States; ^2^ Cardiovascular Disease Initiative, Broad Institute of MIT and Harvard, Cambridge, MA, United States

**Keywords:** endothelial cells, endothelial dysfunction, heart disease, cardiomyocytes, paracrine signaling

## Abstract

The vascular endothelium is a multifunctional cellular system which directly influences blood components and cells within the vessel wall in a given tissue. Importantly, this cellular interface undergoes critical phenotypic changes in response to various biochemical and hemodynamic stimuli, driving several developmental and pathophysiological processes. Multiple studies have indicated a central role of the endothelium in the initiation, progression, and clinical outcomes of cardiac disease. In this review we synthesize the current understanding of endothelial function and dysfunction as mediators of the cardiomyocyte phenotype in the setting of distinct cardiac pathologies; outline existing *in vivo* and *in vitro* models where key features of endothelial cell dysfunction can be recapitulated; and discuss future directions for development of endothelium-targeted therapeutics for cardiac diseases with limited existing treatment options.

## 1 Introduction

The vascular endothelium is one of the largest organs in the body, and is critical for hemostasis, gas and nutrient exchange, and regulation of blood flow. In the heart, endothelial cells (EC) account for a large proportion of non-cardiomyocyte (CM) cells (Litviňuková et al., 2020), supporting the extensive metabolic demands of the myocardium. Dysfunction of the endothelium in cardiac tissue contributes to the manifestations of heart disease. Since first being demonstrated experimentally by Ludmer et al., in 1986, endothelial cell dysfunction (ECD) has primarily been defined as an impairment in the maintenance of vascular tone (Ludmer et al., 1986). In the subsequent decades, ECD has come to encompass a heterogeneous group of endothelial phenotypes characterized by changes in vascular permeability, inflammation, and response to vasoactive stimuli (Bonetti et al., 2003).

Functional cardiac endothelial impairment is most commonly assessed by invasive measurement of coronary flow reserve (CFR) or indirectly by peripheral flow-mediated vasodilation (FMD) upon hyperemic challenge, both of which assess the degree of imbalance between blood supply from the coronary arterial circulation and the metabolic demands of the myocardium (Camici et al., 2015; Alexander et al., 2021). The importance of this paradigm to cardiac health is demonstrated by its well-established prognostic value; for example, a pronounced reduction in FMD is an independent predictor of major adverse cardiac events (MACE) in patients with chronic heart failure (Fischer et al., 2005) and more broadly in meta-analysis of clinical studies of FMD (Matsuzawa et al., 2015). For patients with symptoms of ischemia or angina with no obstruction of the coronary arteries (INOCA/ANOCA), identifying dysfunction of the coronary macro- and/or microvasculature is increasingly recognized as a tool for guiding individual therapeutic strategies (Beltrame et al., 2015). In conditions of ventricular dysfunction such as heart failure with preserved ejection fraction (HFpEF), the degree of CFR reduction is associated with increased heart failure severity (Dryer et al., 2018; Shah et al., 2018). Similarly, decreased responsiveness of myocardial blood flow to dipyridamole-mediated vasodilation was a strong prognostic indicator of worse clinical outcomes in patients with hypertrophic cardiomyopathy (HCM) and was present even in patients with little to no symptoms (Cecchi et al., 2003). The prevalence and chronicity of ECD across many pathologic cardiac states suggests a role for ECD in myocardial dysregulation and maladaptive remodeling beyond impaired oxygen and nutrient delivery. Despite a wealth of clinical findings, definitive mechanistic roles of the endothelium in the onset and progression of disease remain an unsolved question.

Recent studies have substantially advanced the mechanistic understanding of how ECD induces changes in CM function, survival, and growth. For example, endothelium-derived nitric oxide (NO) plays a central role in the regulation of vascular tone and endothelial integrity, but also has demonstrable effects on cardiac contractility (Kelly et al., 1996; Layland et al., 2002) and metabolism (Davidson and Duchen, 2006). The complex paracrine environment of the myocardial niche similarly suggests that the endothelium plays a role in CM homeostasis through secreted factors such as angiotensin-II, endothelin-1, neuregulin-1, and apelin, among others (Colliva et al., 2020). Therefore, chronic disruption of any of these signaling pathways promoted by critical drivers of ECD including pro-inflammatory activation, oxidative stress, endothelial to mesenchymal transition (EndMT), and disturbed shear stress may dynamically impact the function of the myocardium.

While the scope of this piece will be limited to the major drivers of ECD and key paracrine factors, which are most extensively characterized and linked to CM dysregulation, there is emerging data indicating several other paradigms by which CM function may be associated with ECD. For example, EC senescence is triggered by many classical drivers of ECD and may further promulgate cardiac dysfunction (Chen et al., 2022). Dysregulation of factors known to be crucial for vascular and cardiac development may also precipitate ECD and pathologic changes in the adult heart (Sánchez-Duffhues et al., 2018; Souilhol et al., 2020). Further, CM-derived signaling is known to effect reciprocal changes on EC phenotype, however these pathways are less well understood (Colliva et al., 2020).

In this review, we will synthesize the current understanding of EC-CM interactions and explore how the dysregulation of this cellular crosstalk may promote the initiation and progression of cardiac disease. We will also outline existing *in vivo* and *in vitro* models where key features of ECD can be recapitulated and discuss how these models are being used to increase our understanding of endothelium-dependent regulation of cardiac pathophysiology.

## 2 Endothelial dysfunction as a clinical marker of heart disease

### 2.1 Coronary microvascular dysfunction: Definition and clinical assessment

ECD in a cardiac setting is a major constituent of coronary microvascular dysfunction (CMD), as a blunting of the natural regulation of blood supply to the myocardium arises from various stimuli that contribute to both impaired vasodilation and increased vasoconstriction (Camici and Crea, 2007). In many cases, the impacted vasomotor responses are accompanied by adverse remodeling of intramural coronary arteries which may extend across the entire left ventricle (Camici et al., 2012). While CMD is attributable to both endothelium-dependent and endothelium-independent causes, many pathogenic drivers of CMD and ECD may coexist in a given patient and offer valuable context to the impact of ECD on the overall clinical picture. CMD has also shown clinical value as a prognostic indicator, and is associated with a nearly 4-fold increase in mortality and 5-fold increase in MACE as compared to patients with normal perfusion (Gdowski et al., 2020).

CMD is most commonly assessed via measurement of CFR, a ratio of maximal hyperemic flow through the coronary circulation relative to resting flow. Maximal flow is typically achieved by challenge with an endothelium-independent vasodilator such as adenosine, dipyridamole, or regadenoson, although follow-up measurement using endothelium-dependent acetylcholine-mediated vasodilation has recently been shown to provide additive prognostic value (Rahman et al., 2020). The current gold standard for quantification of coronary flow velocity is invasive measurement using a Doppler-tipped guidewire or thermodilution techniques during coronary angiography, or noninvasive assessment via positron emission tomography (PET) in combination with an intravenously administered radiotracer. Several alternate modalities have also been shown to provide accurate and reproducible measurement of CFR or comparable indices, including stress-perfusion cardiovascular magnetic resonance imaging (Zhou et al., 2021), transthoracic Doppler echocardiography (Carbone et al., 2021), and myocardial contrast echocardiography (Kaul, 2012), among others (Camici et al., 2015; Smilowitz et al., 2023). Recently, microvascular resistance reserve (MRR), a novel index of coronary vasodilatory reserve, was found to be advantageous in assessment of vasodilatory capacity in patients with significant epicardial coronary artery disease due to its adjustment for epicardial hemodynamic alterations (Boerhout et al., 2023). FMD of the brachial arteries measured using ultrasonography offers a less intensive, surrogate metric of coronary endothelial function given the close relationship of the coronary and peripheral vasomotor responses (Anderson et al., 1995; Teragawa et al., 2005).

**FIGURE 1:**
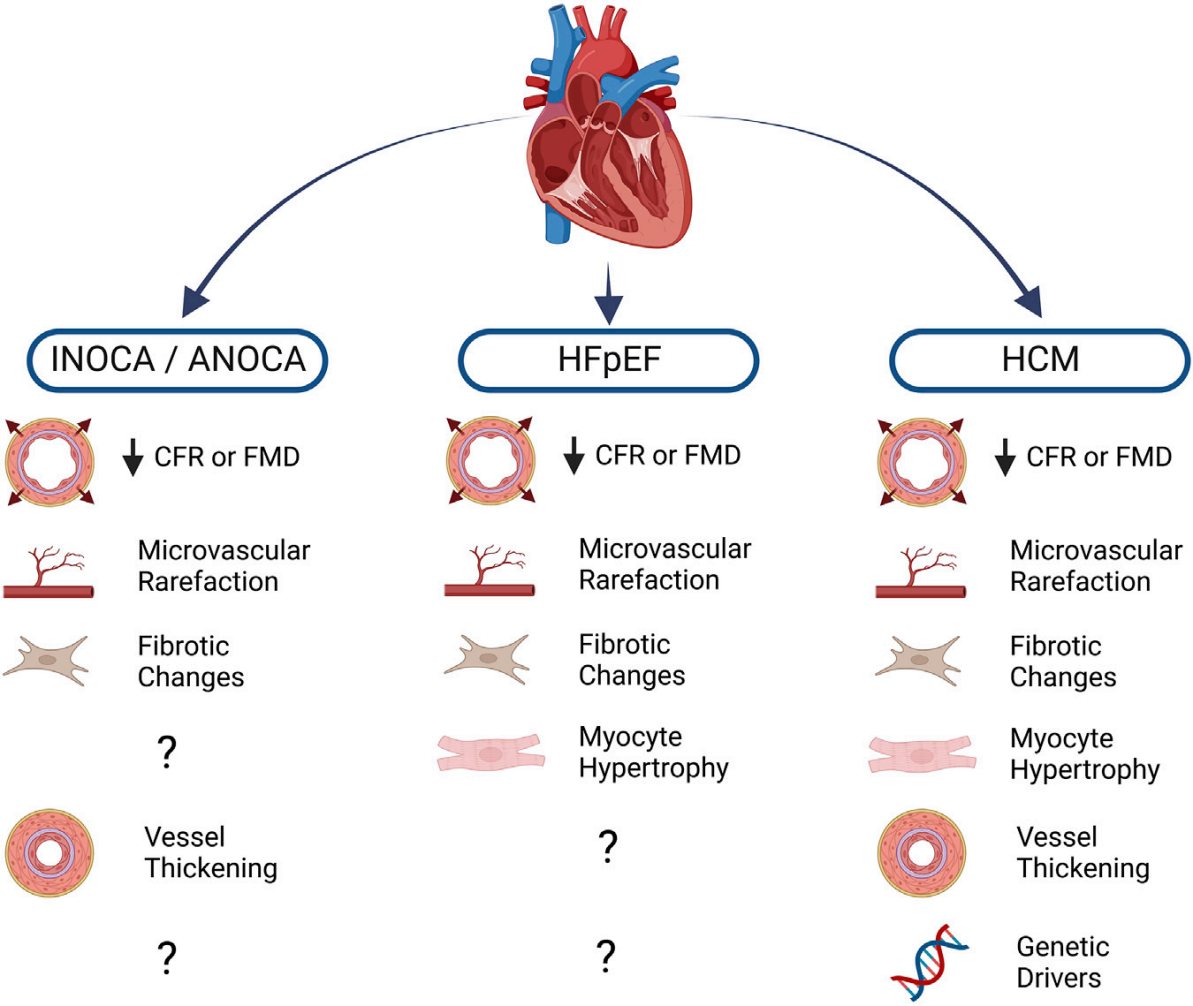
Cardiac diseases and known associations with endothelial dysfunction. Blunted myocardial perfusion as assessed by coronary flow reserve (CFR) or flow-mediated dilation (FMD) reflects impaired vasomotor activity, a major sign of endothelial dysfunction. Structural changes to the microvasculature further compound the loss of myocardial blood flow, often leading to pro-fibrotic and pro-hypertrophic signaling in the diseased heart. Diminished nitric oxide (NO) bioavailability also promotes vascular smooth muscle cell proliferation, leading to thickening of the vascular wall. A number of heritable mutations are well-known drivers of hypertrophic cardiomyopathy (HCM), moreover, population-level genetic analysis has led to investigation of several variants that may be implicated in other conditions. Created with BioRender.com.

While CFR measurement is not performed routinely in a clinical setting, increasing availability of assessment techniques coupled with recent findings indicating CFR’s predictive value in numerous cardiac conditions will likely herald a rise in usage. Along with increased awareness of CMD as a prominent cardiac risk factor, recent trends in clinical data highlight non-obstructive ischemic heart disease (INOCA/ANOCA), hypertrophic cardiomyopathy (HCM), and heart failure with preserved ejection fraction (HFpEF) as three clinical syndromes which have the strongest known associations with CMD and ECD (Gao et al., 2022). Other cardiac conditions in which ECD manifests include valvular disease, infiltrative heart disease, and coronavirus disease 19 (COVID-19) (Crea et al., 2022). While this list is not exhaustive, each of these pathologies is illustrative of the link between ECD and cardiac pathophysiology and serve to highlight areas where further investigation is warranted (Figure 1).

### 2.2 Ischemic heart disease

Up to 40% of patients presenting with chest pain indicative of CAD are found to have normal-appearing coronary arteries upon angiography (Patel et al., 2010). This phenomenon of angina, or demonstrable ischemia, with no obstructive CAD (INOCA/ANOCA) represents a spectrum of clinical syndromes closely linked with CMD, as the presence of coronary ECD can lead to arterial vasospasm causing myocardial ischemia or acute coronary ischemic events. A study of ANOCA patients reported that approximately two-thirds of patients had CMD indicated by measurement of CFR (Sara et al., 2015). Similarly, the ACOVA study found that acetylcholine administration triggered epicardial or microvascular coronary spasm in approximately two-thirds of patients with stable angina and angiographically normal coronaries (Ong et al., 2012). Establishing presence of CMD offers critical prognostic information beyond typical noninvasive testing in patients with ischemic symptoms; the presence of CMD was found to be significantly associated with increased risk of MACE and had a higher prognostic impact than the presence of INOCA (Lee et al., 2022). Given these findings, recent consensus statements recommend coronary flow assessment for all patients presenting with ANOCA/INOCA (Perera et al., 2023).

Current evidence suggests that cardiovascular risk factors induce progressive ECD, thereby blunting physiological and pharmacological vasomotor responses in the primary resistance vessels of the heart and ultimately reducing blood flow to the myocardium. While CMD is most commonly encountered in stable ischemic heart disease, microvascular spasm is also a proposed mechanism underlying myocardial infarction with no obstructive coronary arteries (MINOCA), and patients with MINOCA have been shown to have comparable clinical outcomes to patients with myocardial infarction with obstructive CAD (Choo et al., 2019). Similarly, in a study of 84 patients presenting with ST segment-elevation myocardial infarction (STEMI), macrovascular ECD and/or CMD was detected in the non-culprit region of 93% percent of patients after revascularization (Díez-Delhoyo et al., 2019).

Given that ECD is a hallmark of the early stages of atherogenesis, it is no surprise that there is considerable overlap in the phenotypic spectrum of patients with CMD and patients with CAD. Indeed, alterations in wall shear stresses secondary to reduced coronary flow due to CMD may also further trigger ECD and aggravate atherosclerotic plaque formation (Sabe et al., 2022). Some groups have suggested subclassification of patient populations based on the independent severity of both CMD and CAD, as the degree of CMD carries important implications for the evaluation and selection of appropriate therapeutic strategies for CAD, and *vice versa* (Crea et al., 2014; Taqueti and Di Carli, 2018; Padro et al., 2020).

### 2.3 Hypertrophic cardiomyopathy

Hypertrophic cardiomyopathy (HCM) is a genetic disorder characterized by left ventricular hypertrophy in the absence of sufficient causative hemodynamic stressors (Elliott et al., 2007), and affects approximately 1 in 200 to 1 in 500 individuals in the general population (Maron, 2018). Pathogenic mutations in dozens of genes encoding the CM contractile apparatus have been characterized, however approximately 40% of HCM patients still do not yet have a causative mutation identified (Marian, 2021). Most HCM patients have a relatively benign prognosis, but some exhibit progressive left ventricular dysfunction, heart failure, and an increased risk of sudden cardiac death (Marian and Braunwald, 2017).

In HCM, CMD and accompanying myocardial perfusion deficits result from a combination of increased hemodynamic loading conditions and hypertrophic structural changes (Knaapen et al., 2008). Elevated wall stresses lead to a reduction in coronary flow by compression of intramyocardial arterioles during systole, as well as impaired relaxation during diastole (Raphael et al., 2016). Structurally, myocyte disarray and interstitial fibrosis (Laird et al., 2023) are accompanied by coronary microvascular remodeling and capillary rarefaction (Johansson et al., 2008; Ismail et al., 2014). While this pathological remodeling is likely exacerbated as the hypertrophied tissue outstrips a diminishing oxygen supply, PET measurements of myocardial blood flow (MBF) and CFR in HCM patients revealed presence of CMD in both the hypertrophied septum as well as the non-hypertrophied left ventricular free wall (Camici et al., 2020). Moreover, late gadolinium enhancement was observed in segments of the left ventricle with CMD, supporting the hypothesis that recurring ischemia from CMD eventually precipitates myocyte death and fibrotic remodeling. Prospective analysis of 51 HCM patients revealed that the degree of CMD is a strong prognostic indicator for progressive left ventricular remodeling and clinical deterioration (Olivotto et al., 2006). While these findings implicate CMD in the severity of HCM, no studies to date have investigated the large discrepancies in the disease course of HCM and patient CMD status as they relate to causative mutation or other cardiovascular risk factors.

### 2.4 Heart failure with preserved ejection fraction

Heart failure with preserved ejection fraction (HFpEF) remains as the greatest unmet need in cardiovascular medicine, accounting for over 50% of all heart failure patients and increasing in incidence annually (Tsao et al., 2018). The landmark 2013 proposal by Paulus and Tschöpe suggested an endothelium-centric disease paradigm, wherein an aggregation of cardiovascular comorbidities induce a systemic, low-grade inflammation that triggers CMD (Paulus and Tschöpe, 2013). The resultant disruption of NO production contributes to worsening myocardial stiffness and interstitial fibrotic changes that bring about the HFpEF phenotype. Since 2013, numerous clinical studies have substantiated the link between inflammation, CMD, and HFpEF (Mesquita et al., 2021). Network analysis of 92 biomarkers in a cohort of 1,544 heart failure patients identified processes involved in inflammation, cellular adhesion, and neutrophil degranulation were enriched in the HFpEF cohort as compared to patients with heart failure with reduced ejection fraction (HFrEF) (Tromp et al., 2018). The PROMIS-HFpEF study reported evidence of CMD, defined as CFR <2.5, in 75% of the 202 patients assessed (Shah et al., 2018). Diminished CFR was also associated with markers of heart failure severity such as plasma NTproBNP levels and right ventricular dysfunction. Further, impaired CFR was found to be independently associated with diastolic dysfunction, and patients with both impaired CFR and diastolic dysfunction exhibited a greater than 5-fold increase in hospitalization for HFpEF (Taqueti et al., 2018). These findings advance the theory of a “pre-HFpEF syndrome” in which progressive ischemia secondary to CMD leads to ischemia, CM injury, and myocardial stiffness. In 2021, Paulus and Zile revisited the 2013 proposal in light of new observations linking the HFpEF phenotype to immune infiltration of the myocardium and proinflammatory instigation of CM inducible nitric oxide synthase (iNOS), causing pathologic protein accumulation and fibrotic changes (Paulus and Zile, 2021). The relative contribution of immune response to the characteristic myocardial stiffening in HFpEF is still unknown, but its presence may partially explain the phenotypic differences between HFpEF and other CMD-associated conditions without evidence of diastolic dysfunction, such as INOCA/ANOCA.

**FIGURE 2:**
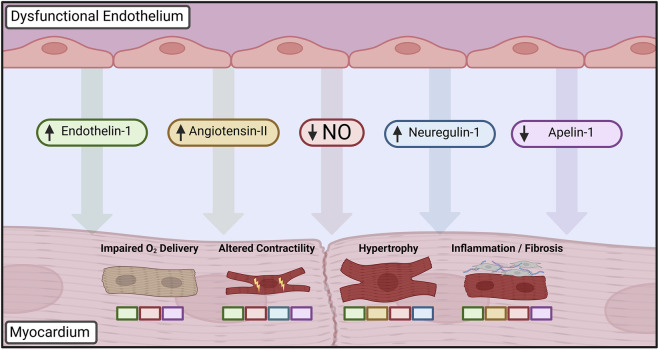
Paracrine mediators dysregulated in dysfunctional endothelium leading to altered cardiomyocyte phenotype. Loss of nitric oxide (NO) bioavailability is central to disruption of cardiomyocyte homeostasis, driving changes in cardiac growth, inflammation, and inotropy in addition to furthering autocrine dysfunction. Upregulation of vasoactive peptides such as endothelin-1 and angiotensin-II leads to further deterioration of vasomotor control in addition to mediating hypertrophic and fibrotic changes. Developmental factors such as neuregulin-1 influence postnatal cardiac growth and contractility, whereas decreased apelin-1 signaling similarly impairs endothelial-cardiomyocyte supporting functions. Colored boxes indicate signaling pathways directly implicated in each cardiomyocyte phenotypic change. Created with BioRender.com.

Given the hallmark presence of ECD in a heterogeneous group of cardiac disease states, there is growing evidence that it may serve as an initiating or aggravating event that is modified by other existing comorbidities. While a definitive causal relationship has thus far been challenging to define experimentally, substantial work has focused on elucidating the aberrant cellular signaling and phenotypic changes in cardiac cells that take place in the context of ECD (Figure 2).

## 3 Mechanisms of cardiac pathophysiology in the context of endothelial dysfunction

### 3.1 Endothelial regulation of cardiomyocyte growth, remodeling, and function

The regulation of vascular tone by NO is central to the current understanding of ECD. NO primarily mediates vasorelaxation by binding to the heme moiety of soluble guanylate cyclase (sGC) in endothelial-adjacent vascular smooth muscle cells (VSMC), initiating the production of the second messenger molecule cGMP. cGMP increases protein kinase G (PKG) activity, ultimately inducing VSMC relaxation (Moncada and Higgs, 2006; Chen et al., 2008; Mónica et al., 2016). Endothelial-derived nitric oxide synthase (eNOS) catalyzes the synthesis of NO from oxygen and L-arginine. Importantly, eNOS is heavily regulated via transcriptional mechanisms, protein-protein interactions, and post-translational phosphorylation (Garcia and Sessa, 2019). The importance of this regulation is readily apparent in a clinical setting as eNOS expression and activation, and thus eNOS catalytic activity, has been shown to be reduced in patients with diabetes mellitus, hypertension, obesity, and chronic inflammation.

The deficits in vasomotor responses characteristic of ECD arise from “uncoupling” of eNOS in settings of cofactor or substrate scarcity, leading to generation of reactive oxygen species (ROS). Substantial levels of cellular oxidative stress can further drive ECD, as superoxide reacts with NO to form peroxynitrite, which is strongly prooxidant and non-vasoactive (Zhang and Shah, 2014).

In the heart, NO is constitutively generated by endothelial eNOS, with some contribution from CM eNOS, neuronal NOS (nNOS), and iNOS in settings of pathological changes. NO has a wide range of downstream signaling actions in CM, the effect of which depends largely on the concentration of cGMP within CM (Andries et al., 1996). NO originating from the coronary microvascular endothelium enhances left ventricular lusitropy via PKG-mediated phosphorylation of Troponin I, thereby decreasing myofilament sensitivity to calcium (Seddon et al., 2007). Biphasic inotropic and chronotropic effects of NO have also been demonstrated, with changes in myocardial contractility and beating rate dependent on concentrations of NO and cGMP (Musialek et al., 1997), an intact endocardial endothelium, and the concomitant presence of cholinergic or adrenergic stimulation (Mohan et al., 1996). A recent preclinical study showed that TNF-α-mediated inflammatory stimulation of cardiac microvascular EC impaired contraction and relaxation of co-cultured CM due to endothelial ROS accumulation and NO scavenging. Interestingly, treatment with empagliflozin restored endothelial NO production and regulation of CM contraction and relaxation by reducing endothelial ROS (Juni et al., 2019). The dose-dependent effects of NO similarly extend to cardiac remodeling, with a baseline anti-hypertrophic effect transitioning to a maladaptive, pro-apoptotic effect in states of high NO or iNOS activation (Wollert and Drexler, 2004). NO has also been shown to modulate CM mitochondrial oxygen consumption via inhibition of cytochrome C oxidase (Davidson and Duchen, 2006), however the cumulative impact of this effect on cardiac metabolism and pathology is unclear.

The NO axis is also involved in driving a number of effector mechanisms within the endothelium which indirectly impact the myocardium. Expression of immune cell adhesion molecules such as VCAM-1 and ICAM-1 are decreased by NO, suggesting that a chronically NO-depleted state may cause increased immune cell recruitment and fibrotic changes (Khan et al., 1996; Riad et al., 2008).

Besides NO signaling, several other factors involved in regulation of vascular tone have been implicated in modulation of CM behavior. Beyond its primary role in the Renin-Angiotensin-Aldosterone-System (RAAS)-governed regulation of blood pressure, the peptide hormone angiotensin-II (Ang-II) has also been linked to ECD in many cardiovascular diseases. Elevated levels of Ang-II are linked to increased arginase activity, thereby reducing available L-arginine for eNOS synthesis of NO (Shatanawi et al., 2011). Ang-II itself is a direct stimulator of hypertrophic CM signaling (Bhullar and Dhalla, 2022) and is also linked to endothelin-1 mediated hypertrophic remodeling (Iwanaga et al., 2001). Ang-II also mediates the release of several pro-fibrotic factors from cardiac fibroblasts, such as TGF-β (Schnee and Hsueh, 2000). Angiotensin receptor blockers (ARBs), one of the most commonly used classes of antihypertensive drugs, have blood pressure-independent improvements in endothelial function (Watanabe et al., 2005), likely due to amelioration of ECD and inflammation.

Endothelin-1 (ET-1) was first described as a potent vasoconstrictive peptide isolated from the vascular endothelium (Yanagisawa et al., 1988). ET-1 has since been found to govern a diverse range of homeostatic mechanisms in the body and is associated with several cardiovascular disease states (Miyauchi and Sakai, 2019). The two known ET-1 receptor subtypes, ET_A_ and ET_B_, have opposing actions that serve to modulate the effect of ET-1. ET_A_ receptors initiate vasoconstriction upon activation, and have also been found to promote inflammation, whereas ET_B_ receptors stimulate endothelial NO and prostanoid release, resulting in increased vasodilation, natriuresis, and reduced inflammation upon activation (Schneider et al., 2007). Given that NO also reduces the release of endogenous ET-1, ECD is often regarded as a disruption in the balance of opposing vasomotor activity from NO and ET-1 (Marasciulo et al., 2006).

With regards to CM signaling, ET-1 has both inotropic and chronotropic effects, and also promotes hypertrophic changes (Cornuault et al., 2022). The production of endothelial-derived ET-1 can be experimentally induced by pro-inflammatory stimuli (Scalera, 2003) and mechanical load (van Wamel et al., 2002). These findings demonstrate paracrine transmission of endothelial disruptions that can alter the phenotype of adjacent CM (Camici et al., 2020). Importantly, ET-1 signaling appears to be necessary for cardiac development and homeostasis, as mice with CM-specific knockout of ET-1 show progressive CM apoptosis, systolic dysfunction, and cardiac dilation (Zhao et al., 2006).

Clinically, plasma ET-1 levels are chronically elevated in settings of LV dysfunction and congestive heart failure (Pousset et al., 1997). Higher baseline ET-1 levels are also independently associated with worse clinical outcomes in heart failure patients (Yeoh et al., 2023) and greater exercise intolerance in patients with HFpEF (Bevan et al., 2021). While ET-1 receptor antagonists present a promising therapeutic avenue, clinical trials have not yet borne out these results (Kirkby et al., 2008), likely in part because endothelin receptors continue to transmit signals from the intracellular space (Archer et al., 2017).

The cardiac endothelium is a major source of secreted proteins with known developmental roles that also influence the function of the adult heart. Neuregulin-1 (NRG-1), produced by cardiac microvascular EC, acts upon ErbB2 and ErbB4 receptors in adult CM with context-dependent effects (Parodi and Kuhn, 2014). Breast cancer therapy with trastuzumab, an anti-ErbB2 monoclonal antibody, was shown to induce dilated cardiomyopathy and heart failure (Seidman et al., 2002), pointing to a role of NRG-1 signaling in cardiac remodeling. *In vitro*, NRG-1 administration increases CM hypertrophy (Garratt et al., 2003), paralleling increased NRG-1 signaling observed in physiological hemodynamic overload during pregnancy (Lemmens et al., 2011). *In vivo*, injection of mice with NRG-1 or overexpression of ErbB4 induces CM cell cycle re-entry and proliferation (Bersell et al., 2009). Notably, activation of VEGFR2 signaling in cardiac ECs induces endothelial release of NRG-1 and other ErbB ligands (Kivelä et al., 2019), suggesting coordination of angiogenesis and CM proliferation during physiologic hypertrophy. Further, NRG-1 appears to have a modulatory role on cardiac contractility, as it exhibits negative inotropic effects. In the context of heart failure ErbB2 and ErbB4 receptors are downregulated (Lemmens et al., 2004), indicating a compensatory attempt to preserve myocardial contractile performance. Early trials of recombinant human NRG-1 in patients with HFrEF showed improvements in patient cardiac output and decreased pulmonary artery wedge pressure (Lim et al., 2015), but larger-scale clinical trials have not yet demonstrated similar efficacy.

The secreted peptide apelin (APLN) is a strong positive inotrope produced by the coronary microvasculature. The APLN receptor APJ is primarily expressed in VSMC and CM and is functionally similar to the Ang-II/Ang-II type I receptor pathway (Chandrasekaran et al., 2008). APLN exerts its powerful inotropic action by increasing the availability of intracellular calcium to enhance myocyte contractility, while also initiating a vasodilatory response (Segers et al., 2018). The combined effect of increased contractility with reduced ventricular preload and afterload implicates APLN as a promising target for treatment of heart failure, along with the observations that tissue levels of APLN are increased in early stages of heart failure but decrease in advanced disease (Chen et al., 2003). Recent animal studies suggest that the APLN/APJ signaling system also directly improves ECD by ameliorating endothelial senescence (Yang et al., 2017), reducing apoptosis and expression of adhesion molecules (Li et al., 2021), and acting as an autocrine cue to enhance angiogenesis and vascular repair following immune-mediated injury (Masoud et al., 2019).

Numerous other endogenously produced cytokines and growth factors in the heart have been identified as mediators of cardiac growth, remodeling, and function without yet being definitively linked to the cardiac endothelium. Comprehensive discussion of these factors, their hypothesized cellular sources, and their paracrine vs. autocrine effects may be found elsewhere (Segers et al., 2018; Segers and De Keulenaer, 2021).

Notably, the close association between cardiac EC and CM also allows for cell-cell signaling through other mechanisms, such as cell junctions, extracellular vesicles, and non-coding RNAs.

Connexins are transmembrane proteins expressed in almost all cardiac cell types, and form connections between neighboring cells in the form of gap junctions. Gap junctions permit passage of water, ions, cellular second messengers, and other small molecules such as polypeptides and miRNA (Rodríguez-Sinovas et al., 2021). Homocellular EC-EC and CM-CM gap junctions are critical for functions such as maintenance of endothelial integrity and coordinated cardiac contractility, respectively (Hesketh et al., 2009; Johnson and Camelliti, 2018). While there is some evidence of heterocellular EC-CM gap junctions (Narmoneva et al., 2004), the putative role of connexins in EC-CM signaling remains largely unknown.

Extracellular vesicles (EVs) are membrane-bound structures carrying bioactive contents including RNAs, proteins, and lipids. EVs are either shed by the endosomal system or originate from the cellular membrane and are released by many constituent cell types in the heart, including CM, EC, VSMC, fibroblasts, and immune cells. Recent evidence suggests that endothelial-derived EVs can have either detrimental or protective effects via promotion of myocyte hypertrophy (Fandl et al., 2023), delivery of proinflammatory cargo, or induction of gene programs involved in vascular maintenance and angiogenesis (Nguyen et al., 2021; Ohayon et al., 2021). Interestingly, EVs originating from EC have been shown to decrease vasorelaxation by reducing endogenous NO production in recipient cells (Brodsky et al., 2004). Conversely, CM-derived EVs have been shown to stimulate eNOS activation in the cardiac endothelium in the context of ischemia-reperfusion injury (Chen et al., 2020). Observed differences in the contents of circulating EVs in various cardiovascular diseases have also been the subject of intense investigation in recent years as a potential prognostic tool (Chong et al., 2019; Mathiesen et al., 2021).

MicroRNA (miRNA) and long noncoding RNA (lncRNA) expressed by EC, CM, and VSMC fine-tune protein expression in response to changing environmental conditions, and in many cases are differentially expressed in patients with cardiovascular diseases (Uchida and Dimmeler, 2015; Wojciechowska et al., 2017; Marinescu et al., 2022). Less is known about the paracrine activity of cardiac miRNAs and lncRNAs, however a recent study by Froese et al. identified two endothelium-secreted lncRNAs termed GADLOR1/2 that are transferred to CM in a GATA2 knockout mouse model of heart failure, whereby they blocked CM stress-induced signaling (Froese et al., 2022). These findings suggest that cell-cell RNA transfer may play a role in propagating cellular disruptions throughout the myocardium.

### 3.2 Impact of EC-CM paracrine signaling on cardiac disease

Characterization of fundamental EC-CM signaling pathways have identified commonalities between symptomatically disparate cardiac diseases. As discussed above, NO dysfunction is a hallmark of CMD and mismatched myocardial oxygen supply-demand in ischemic heart disease, HCM, and HFpEF, among other cardiac conditions (Konst et al., 2020). Further, given its anti-fibrotic effects, decreased NO bioavailability heralds increasing myocardial stiffness and progressive fibrosis (Franssen et al., 2016). Reduced levels of PKG and cGMP in HFpEF cardiac homogenates suggests disruption of the NO axis in HFpEF (Van Heerebeek et al., 2006). Recent genetic studies have also identified variants in eNOS as modifiers for HCM severity (Musicante et al., 2022).

Similarly, plasma levels of ET-1 trend higher in patients with HFpEF (Piatek et al., 2022), whereas they are more than twofold higher in patients with HCM (Hasegawa et al., 1996). Circulating big-endothelin-1, a precursor to ET-1, was recently shown to be associated with all-cause mortality and heart failure progression in HCM (Wang et al., 2017). Genetic variants causing increased circulating levels of ET-1 and higher likelihood of CMD have also been identified (Ford et al., 2020) and are under investigation in the PRIZE trial for INOCA (Morrow et al., 2020; Abraham et al., 2022).

Regulation of angiotensin via angiotensin-converting enzyme (ACE) inhibitors or ARBs has long been a mainstay of optimal medical therapy for patients with obstructive CAD, and initial results for their use in INOCA/ANOCA show promising results (Chen et al., 2002; Pizzi et al., 2004; Pauly et al., 2011). Despite these findings, ACE inhibitors and ARBs are not commonly prescribed in management of INOCA/ANOCA, likely due to a lack of consensus on clinical guidelines (Luu et al., 2022). The recent VANISH trial reported promising results for the use of the ARB valsartan in improving metrics of cardiac structure, function, and remodeling in patients with early-stage HCM, which may be in part attributable to a reduction in susceptibility to hypertrophic and pro-fibrotic signaling in the myocardium (Ho et al., 2021). Similarly, treatment with ACE inhibitors or ARBs was associated with lower all-cause mortality in patients with HFpEF (Lund et al., 2012), suggesting that angiotensin signaling also plays a role in the progression of diastolic dysfunction.

Direct studies of other EC-CM signaling pathways implicated in ECD and CMD such as NRG-1 and APLN are far less common, however dysregulation in both likely plays a role in the translation of ECD and CMD to a cardiac phenotype. Several recent studies have linked lower circulating levels of APLN to obstructive HCM (Yang C. et al., 2022), as well as the increased presence and severity of myocardial fibrosis as measured by late gadolinium enhancement in HCM patients (Zhou et al., 2019).

Further investigation of the interdependence and disruption of these major signaling pathways will likely point to the use of multi-targeted therapeutic strategies to achieve better clinical efficacy as compared to the less pronounced outcomes observed with most single-target treatments to date. Importantly, the homeostatic balance of the major known EC-CM signaling pathways is dependent on the humoral and biomechanical environment of the cardiac endothelium itself. The following section will focus on how initial perturbations of the endothelium initiate ECD.

## 4 Humoral and biomechanical drivers of endothelial dysfunction

### 4.1 Proinflammatory activation

**FIGURE 3:**
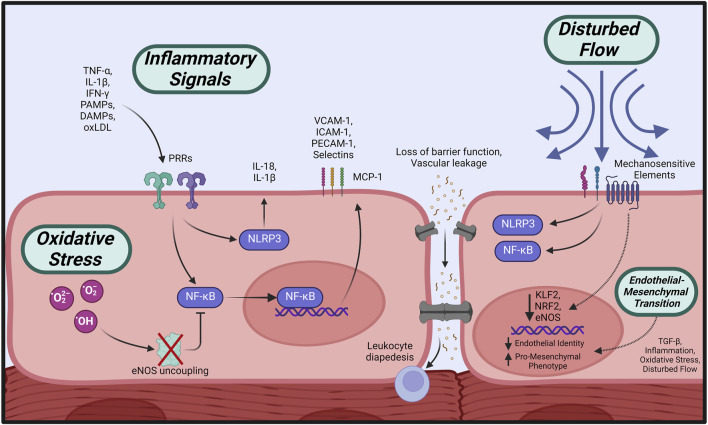
Initiating events leading to endothelial dysfunction. Circulating pro-inflammatory factors cause activation of intracellular effectors such as NLRP3 and NF-κB, which stimulate increased expression of adhesion molecules, loss of barrier function, and propagation of inflammatory signaling. Overabundance of cellular oxidative species leads to eNOS uncoupling and impaired NO bioavailability, in addition to suppressed anti-inflammatory regulation. Disturbed flow across the endothelial surface is transmitted by mechanosensitive elements leading to pro-inflammatory activation. All of the above initiators, in addition to TGF-ß signaling, may induce loss of endothelial identity and a shift towards a profibrotic, contractile phenotype (EndMT). Created with BioRender.com.

The initial response of the endothelium to various inflammatory factors, termed endothelial activation, is central to the cascade of events leading to ECD as characterized by increased vascular permeability, impaired NO signaling, and subsequent vasomotor dysfunction (Theofilis et al., 2021) (Figure 3). Endothelial activation is triggered by inflammatory cytokines (tumor necrosis factor-α (TNF-α), interleukins (ILs) and interferon-γ (IFN- γ)) (Pober and Cotran, 1990; Pober and Sessa, 2007) or binding of pathogen-associated molecular patterns (PAMPs) or damage-associated molecular patterns (DAMPs) to pattern recognition receptors (PRRs) on the endothelial surface (Gong et al., 2020), initiating a series of phenotypic changes in the endothelium.

First, endothelial permeability acutely increases due to formation of focal endothelial gaps, allowing heightened extravasation of plasma and leukocytes (Claesson-Welsh et al., 2021). In situations of chronic inflammation, sustained endothelial activation causes overt remodeling of the microvasculature into a leakier phenotype due to vessel enlargement, proliferation, and changes in junction molecule expression (McDonald, 2001).

Second, increased signaling along innate immune pathways, namely, the NLR family pyrin domain containing 3 (NLRP3) inflammasome and NF-κB, amplifies the proinflammatory response of the endothelium to initiating stimuli. Activation of the NLRP3 inflammasome triggers the release of inflammatory mediators IL-1β and IL-18, which further promote inflammation and oxidative stress (Bai et al., 2020). Similarly, the NF-κB signaling cascade is a principal regulator of the pro-adhesive, pro-thrombotic phenotype in activated endothelium in response to numerous inflammatory cytokines including TNF-α, IL-1β, and IFN- γ (Mussbacher et al., 2019). NF-κB-mediated upregulation of adhesion molecules including VCAM-1, ICAM-1, E-Selectin, and PECAM1, along with monocyte chemoattractant protein (MCP-1) promotes leukocyte rolling, arrest, and diapedesis (Collins and Cybulsky, 2001; Krieglstein and Granger, 2001; Niu and Kolattukudy, 2009). Transendothelial trafficking of monocytes and T-lymphocytes then feeds into neutrophil recruitment with subsequent expression of inflammatory cytokines and ROS generation. This proinflammatory, immune cell-rich milieu of the activated endothelium is a hallmark of atherogenesis.

Finally, inflammation and endothelial activation leads to a pro-thrombotic phenotype due to the upregulation of components of the coagulation cascade including tissue factor (TF) (Borissoff et al., 2011) and von Willebrand factor (vWF) (Dhanesha et al., 2016). NF-κB-mediated downregulation of the anticoagulant thrombomodulin on the endothelial surface in response to inflammatory cytokines further dysregulates the balance between coagulation and fibrinolysis (Sohn et al., 2005).

Under normal conditions, NO acts as a potent suppressor of endothelial activation, inflammation, and thrombosis by s-nitrosylating cysteine residues of a broad spectrum of proteins, thereby reducing their biological activity (Stamler et al., 2001). Moreover, endothelial NO has been shown to play a role in maintenance of cytoskeletal architecture and junctional permeability (Di Lorenzo et al., 2014). NO also has a regulatory role in leukocyte adhesion and platelet aggregation via inhibition of NF-κB signaling (Spiecker et al., 1998) and activation of cGMP-dependent protein kinase, respectively (Wang et al., 1998). In conditions of elevated oxidative stress, uncoupling of NOS and the resulting NO deficit drives endothelial activation.

### 4.2 Oxidative stress

Oxidants such as superoxide anion, hydrogen peroxide, and hydroxyl radical are normal products of aerobic metabolism and catabolic cellular processes which are countered by endogenously produced enzymatic and nonenzymatic antioxidants (Ray et al., 2012). The detrimental effects of ROS arise when their production surpasses the antioxidant capacity of the cell. NADPH oxidases (NOXs) are chiefly implicated in initiation of endothelial oxidative stress as they are the only family of enzymes capable of directly generating superoxide without an external ROS source. Endothelial NOX1, NOX2, and NOX5 activity has been linked to vascular disease in both patients and animal models (Lassègue and Griendling, 2010; Drummond and Sobey, 2014). Additional sources of ROS generation in the endothelium include monoamine oxidase, xanthine oxidase, cyclooxygenase, lipoxygenase, and mitochondrial metabolism (Shaito et al., 2022).

As discussed previously, excess oxidative stress in the endothelium primarily impacts NO production, both by direct conversion of superoxide anion and NO to peroxynitrite (Liaudet et al., 2009) as well as through ROS-induced oxidation of required eNOS cofactors such as tetrahydrobiopterin (BH_4_) or L-arginine (Alp and Channon, 2004). Importantly, uncoupling of eNOS by reduced BH_4_ bioavailability also leads to increased superoxide formation by eNOS itself, furthering inhibition of endothelial NO signaling (Santhanam et al., 2012). Ultimately, ROS-induced dysfunction of the NO axis initiates pathologic changes to the vasomotor response characteristic of ECD.

### 4.3 Endothelial-to-mesenchymal transition

The phenotypic change of endothelial cells towards a mesenchymal cellular state is known as endothelial to mesenchymal transition (EndMT). EndMT is critical for developmental processes (Bischoff, 2019) and has been implicated in several cardiovascular pathologies (Evrard et al., 2016; Souilhol et al., 2018; Kovacic et al., 2019). This cellular transition involves the loss of markers of endothelial identity (e.g., VE-cadherin, eNOS) and the increased expression of mesenchymal markers (e.g., alpha smooth muscle actin, calponin). Moreover, some functional changes have also been documented during this transition. For example, endothelial cells undergoing EndMT display a reduced ability to uptake LDL (Moonen et al., 2010), but an increase in cellular contractility and in their capacity for production of extracellular matrix components (Krenning et al., 2008; Moonen et al., 2010). Notably, the extent of the molecular and functional changes needed to define EndMT progression remains to be fully defined. Nevertheless, this transitional cellular state can be considered a manifestation of endothelial cell dysfunction. Several factors have been shown to promote EndMT, including TGF-ß (Cooley et al., 2014; Pardali et al., 2017), pro-inflammatory cytokines (Xiong et al., 2018), increased oxidative stress (Evrard et al., 2016), and disturbed flow (Moonen et al., 2015). Importantly, in the context of cardiac disease, it is not clear how EndMT promotes distinct pathogenic processes. The best characterized role of EndMT in cardiac disease is the generation of pro-fibrotic cells (Zeisberg et al., 2007), which are drivers of cardiac fibrosis (Widyantoro et al., 2010) leading to multiple pathologies including HFpEF (Paulus and Tschöpe, 2013; Tona et al., 2021).

### 4.4 Biomechanical disturbances

The hemodynamic forces generated by the flow of blood are a key influence on endothelial structure and function. The observation that the earliest atherosclerotic lesions characteristically form in regions of arterial bifurcation, oscillatory shear stress, and turbulent flow has long been appreciated, in contrast to uniform, tubular regions which are relatively protected from atherogenesis (Glagov et al., 1988). More recently, these observations have been expanded by characterization of the substantial differences in endothelial phenotype between areas of “atheroprotective” vs. “atheroprone” flow (Gimbrone and García-Cardeña, 2016).

Signal transduction of shear stresses experienced by the endothelium are initiated by mechanosensitive elements including integrins, G-protein coupled receptors, cytoskeletal sensors, junctional proteins, and the endothelial glycocalyx, among others (Fang et al., 2019). These signals are then integrated into functional changes via gene expression and post-transcriptional events. Several transcriptional regulators have been identified in the control of downstream endothelial responses to shear, the most relevant of which are Krüppel-like factor 2 (KLF2) and NF-E2-related factor 2 (NRF2). KLF2 is a critical regulator of the atheroprotective endothelial phenotype whose expression is initiated by activation of shear-responsive MEKK3/MEK5/ERK5/MEF2 signaling (Parmar et al., 2006). KLF2 regulates transcriptional pathways involved in vascular tone, thrombosis, inflammation, and angiogenesis, and is notably downregulated in regions of disturbed flow, such as the aortic arch (Nakajima and Mochizuki, 2017). Interestingly, knockout of KLF2 paralogues in zebrafish led to loss of integrity and extrusion of the myocardial wall which was prevented by transgenic expression of KLF2 in the endothelium, implicating it as a key regulator of endothelial-myocardial development (Rasouli et al., 2018). NRF2 is activated by both laminar shear stress (Warabi et al., 2007) or cellular oxidative stress (Nguyen et al., 2009) and plays an important role in the antioxidant response by upregulating expression of enzymes such as glutathione S-transferase A2 (GSTA2) and NAPDH: quinone oxidoreductase 1 (NQO1).

Laminar shear stresses also stimulate an anti-thrombotic phenotype owing to increased release of prostacyclin (Frangos et al., 1985) and upregulated thrombomodulin expression (Takada et al., 1994). Concurrent abrogation of inflammatory activation under laminar shear stress occurs due to inhibition of TNF-α-stimulated expression of adhesion molecules CX3CL1, VCAM-1, and ICAM-1. These effects appear to be mediated by flow-sensitive miRNA miR-29b-3p blocking NF-κB signaling (Pu et al., 2022). Similarly, a recent whole-transcriptome study of EC subjected to laminar shear stresses demonstrated an anti-inflammatory effect mediated by nuclear translocation of high-mobility group protein (HMGB1) and activation of autophagy pathways (Meng et al., 2022). Endothelial proliferation also decreases under laminar shear via sustained p53 upregulation, thereby inhibiting cyclin-dependent kinase (Lin et al., 2000). Further, eNOS expression is upregulated (Woodman et al., 2005) and phosphorylation at Ser1177 is enhanced (Dimmeler et al., 1999), leading to increased NO production and a flow-dependent vasodilatory effect. As previously discussed, adequate bioavailable NO prevents endothelial activation by inhibiting expression of MCP-1 and VCAM-1, reducing lipid oxidation and platelet aggregation, and preventing apoptosis (Pan, 2009).

Conversely, disturbed or oscillating shear stresses induce activation of pro-atherogenic gene programs implicated in ECD. Conditions of low or oscillating shear significantly upregulates NF-κB activation, which then promotes release of MCP-1 along with upregulated expression of adhesion molecules such as P-selectin and ICAM-1 to induce leukocyte recruitment (Mohan et al., 1997; Hsiai et al., 2003). The preferential expression of the transcription factor TWIST in athero-susceptible regions of the mouse aorta has also been documented to promote atherogenesis via the induction of proliferative and pro-inflammatory pathways (Mahmoud et al., 2016). Oscillatory flow was also found to induce formation of the NLRP3 inflammasome in mouse aortas, accelerating atherogenesis under hyperlipidemic conditions (Xiao et al., 2013). Chromatin immunoprecipitation analysis of human umbilical vein EC (HUVEC) indicated that oscillating shear stress activates the HIPPO pathway via its master mediator YAP/TAZ (Bondareva et al., 2019), which is associated with endothelial proliferation, inflammation, and atherogenesis (Wang et al., 2016). Several animal models of acutely disturbed flow demonstrate downregulation of KLF2 and eNOS alongside activation of transcription factors NF-κB and AP-1, increased expression of MCP-1, and accelerated atherogenesis (Nam et al., 2009; Juncos et al., 2011).

Mechanical regulation of endothelial phenotype has also recently been linked to a repertoire of flow-responsive miRNAs shown to modulate inflammatory response, cytoskeletal control, and other flow-sensitive factors such as KLF2, among other pathways. Similarly, changes in shear stresses experienced by the endothelium have been shown to drive epigenetic effectors such as DNA methylation, histone modification, and expression of lncRNAs. Comprehensive discussion of these systems of mechanotransduction may be found elsewhere (Ku et al., 2019; Herrera and Schwartz, 2022).

### 4.5 Cardiovascular risk factors as drivers of endothelial dysfunction

Many cardiac diseases, including those discussed in this review, have come to be understood as syndromes arising from a confluence of many coexisting and modifying risk factors. This paradigm explains why many clinical trials targeting individual pathways in diseases such as INOCA/ANOCA, HCM, and HFpEF have yielded neutral or negative results. Many classical risk factors for cardiovascular disease have been connected to the onset and progression of ECD by way of increased inflammation, a greater burden of oxidative stress, or disrupted hemodynamics.

In terms of inflammatory activation, numerous studies show an association between NLRP3 activity and conditions involving ECD such as diabetes (Gora et al., 2021), hypertension (De Miguel et al., 2021), obesity (Rheinheimer et al., 2017), and atherosclerosis (Duewell et al., 2010) (all of which are also comorbidities for the diseases discussed in this review). Many of these comorbidities are also associated with increased presence of DAMPs and other inflammatory mediators that participate in endothelial activation. For example, HMGB1 is upregulated in patients with diabetes and binds to Toll-like receptors (TLRs) and receptors for advanced glycation end products (RAGEs) to activate NF-κB (Behl et al., 2021). Similarly, excess adipose tissue in obesity releases proinflammatory cytokines and chemokines such as IL-6, IL-1β, TNF-α, and MCP-1 (Kwaifa et al., 2020). Hypercholesterolemia triggers vascular inflammation via NF-κB activation by oxidized LDL (oxLDL), which leads to an increase in iNOS activity and oxidative stress alongside downregulation of eNOS (Gliozzi et al., 2019).

Inflammation and oxidative stress are closely linked drivers of ECD and frequently present concurrently. Increased oxidative stress has well-documented associations with cardiovascular risk factors such as hypertension (Touyz et al., 2020), hypercholesterolemia (Singh et al., 2017), diabetes, obesity, and smoking (Niemann et al., 2017). In addition to its proinflammatory effects, diabetes has been extensively linked to ROS-mediated ECD. Increased production of advanced glycation end products (AGE) and protein kinase C activation in hyperglycemic conditions stimulates ROS production, eNOS uncoupling, and reduces endothelial eNOS expression (Giacco and Brownlee, 2010). Obesity is similarly associated with a greater baseline burden of oxidative stress due to various systemic disturbances such as increased endothelial NOX expression (Silver et al., 2007) and decreased endogenous antioxidant levels (Olusi, 2002). NOX overexpression is also linked to hypertensive conditions by derangements in circulating vasoactive factors including Ang-II, ET-1, and aldosterone (Touyz et al., 2020). With regards to hypercholesterolemia, oxLDL binding to its receptor LOX-1 increases ROS generation via initiation of NOX activity on the cell membrane. LOX-1 signaling also activates arginase II, further antagonizing NO formation by competing with eNOS for its substrate L-arginine (Gradinaru et al., 2015).

Associations between cardiovascular risk factors and changes to the hemodynamic environment of the endothelium are less clearly defined, however there is evidence to suggest that many cardiac comorbidities influence the capacity of the endothelium to respond to shear stresses, likely due in part to phenotypic disruptions caused by inflammatory activation and oxidative stress. Deterioration of the endothelial glycocalyx in diabetes likely compromises surface mechanosensing and furthers ECD (Dogné et al., 2018). Obesity-induced impairment of flow-mediated dilation has been linked to the loss of flow sensitivity of endothelial inward-rectifying K+ (Kir) channels, which may also be related to thinning of the glycocalyx (Fancher et al., 2020). A study of endothelial function in 500 individuals showed that the loss of endothelium-dependent vasodilation was found to be associated with the same risk factors that are well-established drivers of atherogenesis including hypertension, hypercholesterolemia, cigarette smoking, older age, and male sex (Celermajer et al., 1994). Aging-related reduction in endothelial shear stress was also found to be an independent predictor for atherosclerotic development, highlighting the importance of mechanical stimuli in maintaining endothelial function (Carallo et al., 2016). Accordingly, exercise-induced increases in blood flow have endothelial-specific benefits due to the effects of flow-dependent vasodilators and progressive vascular remodeling (Niebauer and Cooke, 1996; Tinken et al., 2010).

Despite the growing understanding of the associations between ECD and many cardiac diseases, there is an increasing need for relevant models of ECD and EC-CM signaling that better interrogate the key disruptions that drive ECD and CMD into an overt cardiac phenotype.

### 4.6 Current clinical strategies targeting endothelial dysfunction

Despite compelling evidence suggesting a central role of the endothelium in several cardiac diseases, therapeutic strategies targeting ECD and CMD in ischemic heart disease, HCM, and HFpEF remain scant.

At present, there are no formalized guidelines for treatment of the clinical spectrum of CMD in the context of ischemic heart diseases. Therapeutic approaches centering on restoration of appropriate endothelial vasomotor function are relegated to management of comorbidities given the highly multifactorial nature of CMD and its overlap with atherosclerosis. Optimized medical therapy including anti-platelet treatment, anti-ischemic therapy, and lipid management is associated with demonstrable reduction in angina and ischemia in patients with stable CAD (Boden et al., 2007; Shaw et al., 2008). However, no studies have yet demonstrated how these beneficial effects are stratified based on severity of underlying CMD.

Proangiogenic therapies to overcome myocardial perfusion deficits in myocardial ischemia have been the focus of several clinical trials based on promising preclinical data, but have not yet made further progress due to a lack of clear therapeutic efficacy (Van Der Laan et al., 2009; Mitsos et al., 2012). The lack of significant therapeutic benefit is likely due to these treatments only involving a single growth factor or gene therapy. These findings highlight the need for more sophisticated models of the heterocellular myocardial niche in order to better define the combination of developmental cues necessary for revascularization of the post-ischemic heart. Similarly, many small-cohort studies have demonstrated that mesenchymal stem cell (MSC)-based therapies encourage endothelial progenitor cell proliferation and modestly improve cardiac metrics, but as of yet no trials have demonstrated a greater benefit than traditional pharmacologics (Poomani et al., 2022). Further, large-scale randomized clinical trials of MSC and other cell-based therapies for ischemic heart disease have yet to be conducted (Bairey Merz and Marbán, 2022). A number of strategies for endothelium-dependent cardioprotection to attenuate ECD and ischemia-reperfusion related injury following MI are also under active investigation including exercise preconditioning, mitochondrial transplantation, and pharmacologic repurposing (Herrera-Zelada et al., 2021). Such areas of investigation would similarly benefit from an increased understanding of EC-CM crosstalk and more sophisticated models of the perfusable myocardium.

While there have been significant advances in the understanding of the endothelium in HCM progression and pathogenesis, there are no clinical trials assessing endothelium-targeted therapies to date. Clinical trials for late sodium current inhibitors ranolazine and eleclazine have also not yielded any evidence of efficacy (Elliott, 2018). The relative rarity of HCM adds to the difficulty of conducting suitably powered randomized controlled trials necessary for revealing small therapeutic effects in new candidate drugs. Recently emerging model systems such as genetically modified human induced pluripotent stem cell-derived cardiomyocyte (hiPSC-CM) lines promise better elucidation of the highly variable disease phenotype and penetrances characteristic of HCM (Mosqueira et al., 2019).

Clinical trials of therapies targeting ECD in HFpEF have thus far yielded disappointing results as well. Attempts to increase NO bioavailability via administration of nebulized inorganic nitrite (INDIE-HFpEF Trial) (Borlaug et al., 2018) or isosorbide mononitrate (NEAT-HFpEF Trial) (Redfield et al., 2015) yielded no significant improvements in the treatment groups. Other attempts to target the NO axis, including stimulation of soluble guanylate cyclase (sGC; VITALITY-HFpEF (Armstrong et al., 2020), CAPACITY-HFpEF (Udelson et al., 2020), and SOCRATES-Preserved Trials (Pieske et al., 2017)) and inhibition of phosphodiesterase-5 to increase cGMP levels (RELAX Trial) (Redfield et al., 2013) have resulted in no improvements in patient clinical status.

To date, apart from lifestyle reduction of cardiovascular risk factors, the sodium/glucose cotransporter-2 inhibitor (SGLT2i) empagliflozin and the glucagon-like peptide receptor agonist (GLP-1 RA) semaglutide are the only therapeutics with a demonstrated improvement in cardiovascular metrics and/or outcomes in multi-center, randomized, double-blind, placebo-controlled trials in patients with HFpEF. The EMPEROR-Preserved Trial showed a reduction in cardiovascular mortality and heart failure-related hospitalization in patients with HFpEF after empagliflozin treatment, regardless of diabetic status (Anker et al., 2021). While the cardiac mechanism of action of gliflozins is still undetermined, recent preclinical evidence suggests a pleiotropic effect on the endothelium. In a nanoplastics-induced model of EC senescence and dysfunction, enavogliflozin increased endothelial eNOS levels, improved vasodilatory capacity, and reduced ROS generation in isolated porcine coronary artery (Dhakal et al., 2023). Similarly, in a murine model of ischemia-reperfusion induced CMD, dapagliflozin treatment was shown to protect endothelial barrier function, eNOS activity, and angiogenic capacity (Ma et al., 2022). These results suggest that SGLT2i-mediated amelioration of ECD may be in part responsible for the observed cardiac improvements in HFpEF. More recently, semaglutide treatment of patients with HFpEF and obesity in the STEP-HFpEF Trial demonstrated larger reductions in heart failure-related symptoms and improvements in exercise function as compared to the placebo group (Kosiborod et al., 2023). While some degree of improvement is attributable to concurrent weight loss in the semaglutide treatment group, decreased CRP and NT-proBNP levels suggest that additional antiinflammatory and hemodynamic effects of GLP-1 RA treatment may be involved.

While the most prominent clinical approaches to amelioration of ECD in heart disease are discussed above, extensive discussion of small-cohort and currently ongoing clinical trials and their outcomes have been recently summarized elsewhere (Tsigkou et al., 2023). The underwhelming results of most current therapeutic strategies directly targeting the endothelium for heart disease reflect the need for better preclinical models of ECD and CMD. Despite well-established associations of ECD with cardiac risk in humans, the complex interplay of endothelial dysregulation and cardiovascular risk factors over years or decades in the adult heart is not yet understood. *In vivo* and *in vitro* systems that can adequately capture the development of ECD and cardiac pathophysiology represent a powerful tool for identifying and testing novel therapeutics with better probability of clinical efficacy.

## 5 Current models of endothelial dysfunction in cardiac development and disease

### 5.1 Animal models of EC-CM signaling and endothelial dysfunction

**FIGURE 4:**
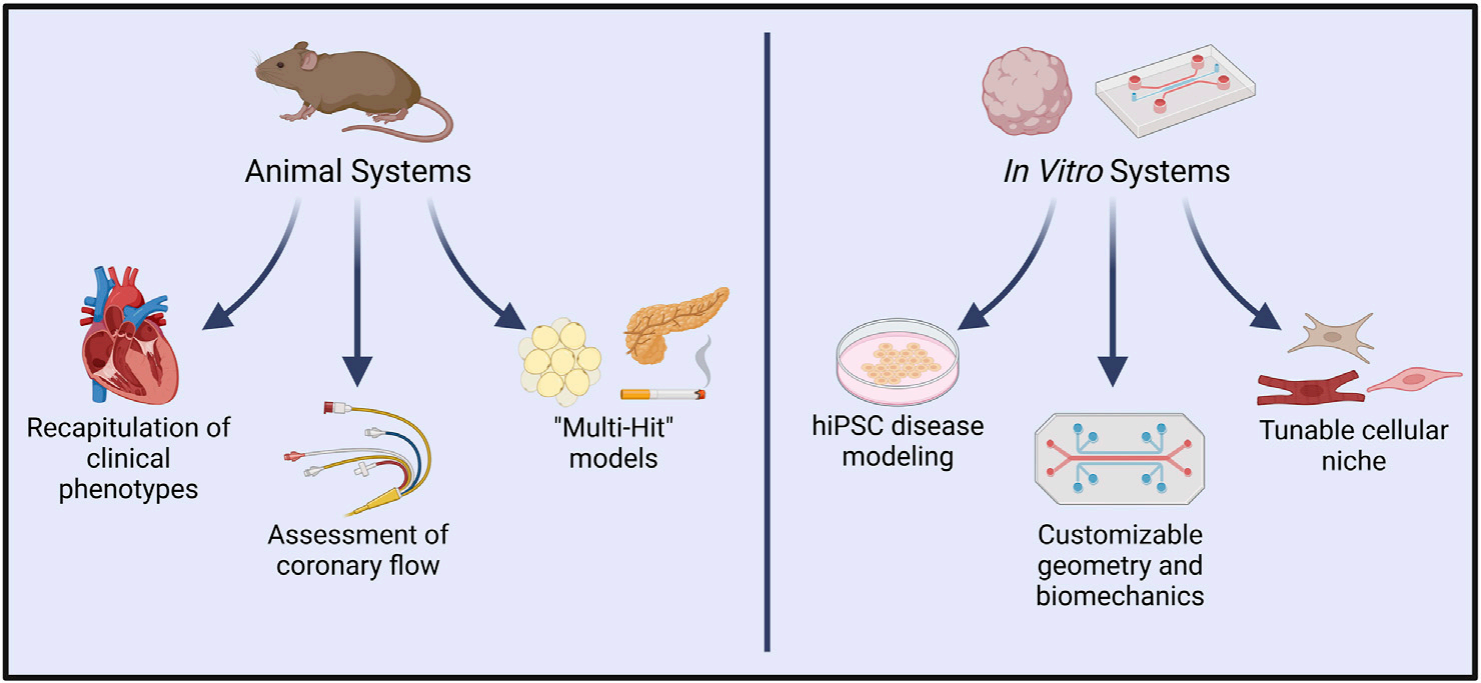
*In vivo* vs. *in vitro* systems for modeling endothelial dysfunction and complex cardiac disease. Animal models allow experimental induction of clinical symptoms that closely resemble those seen in cardiac conditions such as INOCA/ANOCA, HCM, and HFpEF. Resultant functional changes such as diminished coronary flow are also readily quantified in such models. In recent years, “multi-hit” models of heart disease have more precisely captured the influence of comorbidities typically present in human pathologies. Conversely, *in vitro* platforms permit modeling with human cells, and have already shown great utility in hiPSC-based modeling of genetic diseases. Extensive customizability of *in vitro* systems allows controlled interrogation of cell-cell signaling, spatial organization, and hemodynamic environments. Introduction of other cardiac cell types, such as endothelial cells, fibroblasts, neurons, and immune cells promises to further recapitulate native human pathophysiology. Created with BioRender.com.

Mouse models of ECD are uniquely advantaged for modeling pathway-specific endothelial regulation of cardiac function due to the ease of developing genetically modified mice (Figure 4). Assessment of cardiac phenotype in mice with endothelial-specific genetic modifications may provide valuable insights into EC-CM crosstalk. Indeed, several such studies have identified endothelial genetic modifications that are sufficient to drive heart failure (Cornuault et al., 2022).

Huang et al. provided one of the first demonstrations of the importance of NO in endothelial regulation of blood pressure in a mouse knockout of eNOS, in which animals developed severe hypertension and loss of acetylcholine-induced vasodilation (Huang et al., 1995). Similarly, platelet endothelial cell adhesion molecule (PECAM-1) has been described to have mechanosensory functions that regulate eNOS activity in response to shear stresses (McCormick et al., 2011). Mice with endothelial knockout of PECAM-1 exhibit LV dilation and systolic dysfunction along with elevated endothelial NO, ROS, and NRG-1 signaling. Importantly, blocking NRG-1 in PECAM^−/−^ mice significantly improved cardiac function, implicating PECAM-1 as a regulator of cardiac function via paracrine NRG-ErbB2 signaling (McCormick et al., 2015). Conversely, sustained endothelial activation of β-catenin suppressed endothelial NRG-1 expression and cardiac ErbB2/ErbB4 signaling, resulting in progressive cardiac dysfunction that was rescued by exogenous NRG-1 treatment (Nakagawa et al., 2016).

Other endothelial knockout studies have identified the importance of endothelial regulation of the CM metabolic state in disease onset. Inhibition of Notch signaling via endothelial deletion of Rbpj has been shown to impair fatty acid trafficking across the cardiac endothelium and stimulate cardiac angiogenesis, leading to cardiac hypertrophy and failure (Jabs et al., 2018). Similarly, disruption of the endothelial receptor tyrosine kinase EphB4 induces pathological cardiac remodeling resembling dilated cardiomyopathy due to accompanying disruption of capillary integrity and lipid trafficking (Luxán et al., 2019).

Interesting, vascular endothelial deletion of Cx43 induces hypotension and bradycardia in mice owing to elevated plasma NO and angiotensins, suggesting a role of Cx43 gap junctions in NO signaling (Liao et al., 2001). Given the existence of heterocellular EC-CM Cx43 gap junctions within the myocardium, further investigation of CM phenotype in such a model is warranted (Narmoneva et al., 2004).

Other preclinical studies have successfully induced cardioprotective effects in mouse models of cardiac dysfunction, hinting at potential therapeutic targets in the endothelium. For example, disruption of endothelial mineralocorticoid signaling via knockout of Nr3c2 (Jia et al., 2015) or Scnn1a (Sowers et al., 2020) prevented development of diastolic dysfunction and reduced inflammatory and oxidative burden in HFD mice. Prevention of leukocyte recruitment during endothelial activation by endothelial ICAM1 knockout was shown to prevent cardiac dysfunction and fibrosis in a thoracic aortic constriction (TAC) mouse model of pressure overload, representing proinflammatory endothelial activation as a potential target for heart failure (Salvador et al., 2016). Direct endothelial regulation of cardiac fibrosis has also been demonstrated via endothelial overexpression of FOXP1 (Liu et al., 2019) or disruption of Ets1 (Xu et al., 2019), both of which prevented Ang-II-mediated cardiac hypertrophy and fibrosis.

Genetic alterations specific to CM similarly serve to elucidate the delicate homeostatic control of cardiac growth and remodeling. CM-specific overexpression of placental growth factor (PlGF) stimulates myocardial angiogenesis and increases endothelial release of NO, resulting in myocardial hypertrophy via degradation of RGS4, a regulator of G-protein signaling involved in the CM hypertrophic response (Jaba et al., 2013).

Animal models have also been critical in confirming mechanistic drivers of CMD in the context of comorbid metabolic derangements (Sorop et al., 2020). For example, Otsuka Long-Evans Tokushima Fatty (OLETF) and Goto-Kakizaki (GK) rat models exhibit hypercholesterolemia and hyperglycemia from insulin resistance that progresses to impaired insulin secretion. OLETF and GK rats exhibit impaired endothelium-dependent vasodilation very early in the disease process (Mishra et al., 2014; Brown et al., 2018), whereas other rat models of obesity and insulin resistance such as the obese Zucker rat (OZR) and high-fat or Western diet (HFD/WD) models maintain coronary vasodilation in response to acetylcholine, likely due to compensatory mechanisms at early stages of ECD (Jebelovszki et al., 2008; Climent et al., 2017). Importantly, all of these model lines exhibit increased coronary or cardiac oxidative stress which appears to precede changes in cardiac flow (Santos et al., 2003; Katakam et al., 2005; Kajikuri et al., 2009; Ballal et al., 2010).

Some of the earliest murine models of ECD include the leptin receptor deficient (db/db) and leptin deficient (ob/ob) mice, which exhibit polyphagia, obesity, and type 2 diabetes mellitus due to loss of leptin signaling (Tran et al., 2020). Several studies of db/db mice report endothelium-dependent vasomotor dysfunction (Bagi et al., 2004; Gao et al., 2007; Lee et al., 2017). Similarly, ob/ob mice display reduced hyperemic coronary flow and CFR (Westergren et al., 2015; Adingupu et al., 2019). ApoE knockout mice are commonly used as a model of atherogenesis, however they also demonstrate coronary ECD due to oxLDL-mediated impairment of NO (Xu et al., 2007).

Larger animal models also offer valuable insight into development of CMD, as PET studies and invasive techniques for measurement of coronary flow are more feasible. Canine and porcine models also offer better approximations of the size and coronary anatomy of the human heart. Canines fed with HFD exhibit increased constriction of the coronary microvasculature due to increased Ang-II (Zhang et al., 2005), sympathetic nervous activation (Dincer et al., 2006), and ET-1 (Knudson et al., 2006). Similarly, domestic swine with streptozotocin-induced diabetes fed with HFD for 10 weeks showed evidence of CMD characterized by impaired bioavailability, with a compensatory reduction in ET_A_ receptor response to ET-1-mediated vasoconstriction (Van Den Heuvel et al., 2012). In a follow-up experiment at 15 months, endothelial-dependent vasodilation had normalized, but ET_B_ receptor-mediated vasoconstriction had become markedly enhanced along with significant structural alterations to the coronary vascular bed, underscoring the significance of longitudinal studies assessing duration of exposure to various cardiac risk factors (Sorop et al., 2016).

### 5.2 Animal models of endothelial dysfunction in INOCA/ANOCA, HCM, and HFpEF

Patient studies of INOCA/ANOCA are limited due to diagnosis typically only occurring after symptoms establish, thus making it challenging to assess the contribution of individual risk factors in the structural microvascular alterations and progressive impairment of CFR over time. There are currently no animal models of INOCA/ANOCA, although the development of CMD and structural remodeling of the coronary microvasculature in many of the studies discussed above serves as a useful model of the early perturbations in nonobstructive cardiac ischemia and has hinted at novel treatment options. Indeed, treatment of HFD-fed rats with the ACE inhibitor captopril ameliorated bradykinin-induced vasodilation, paralleling findings in isolated coronary arterioles from obese patients (Feher et al., 2013). These findings are borne out by clinical evidence showing that ACE inhibition improves CFR in patients with diabetes (Kawata et al., 2006) and INOCA/ANOCA (Chen et al., 2002; Pizzi et al., 2004; Pauly et al., 2011).

Animal models for assessing the sarcomeric protein variants linked to HCM take advantage of naturally occurring cardiomyopathies (e.g., domestic cats) (Longeri et al., 2013), however transgenic models enable more thorough molecular analysis of causative mutations (Duncker et al., 2015). Given the growing understanding of the role of ECD and structural changes to the coronary microvasculature in HCM phenotype and disease severity, there is a need for adequate model systems beyond assessment of putative defects in the CM contractile apparatus.

Most preclinical HFpEF models do not fully meet clinical diagnostic criteria for HFpEF; to date, only a handful of combinatorial models have been shown to closely resemble human HFpEF (Withaar et al., 2021a). Schiattarella et al. demonstrated that mice fed with a high-fat diet (HFD) and treated with L-NAME developed pulmonary congestion, diastolic dysfunction, cardiac hypertrophy with fibrosis, and exercise intolerance, whereas treatment with HFD or L-NAME alone only elicited a partial phenotype. HFD + L-NAME mice also developed a systemic inflammatory state and concomitant activation of iNOS, paralleling findings in human HFpEF hearts and suggesting iNOS as a key mediator of HFpEF development (Schiattarella et al., 2019).

Another recently reported model involved aged (18–22 months) mice given HFD and Ang-II infusion, closely recapitulating the common cardiometabolic HFpEF phenotype characterized by advanced age, obesity, type 2 diabetes mellitus, hypertension, and female sex. This Aging + HFD + Ang-II mouse model closely matches the clinical features of HFpEF including pulmonary congestion, diastolic dysfunction, atrial enlargement, concentric LV hypertrophy, and hypertension. Treatment with the glucagon-like-peptide-1 receptor agonist (GLP-RA) Lira improved cardiac function and attenuated adverse cardiac remodeling, demonstrating GLP-1 RAs as a potential therapeutic target for HFpEF (Withaar et al., 2021b). These data are further supported by recent findings that GLP-1 RA treatment can reduce ECD via antioxidant, anti-inflammatory effects (Helmstädter et al., 2020). Similarly, Deng et al. developed a 3-hit model of HFpEF in aged (16 months) mice given HFD and 3 months of the mineralocorticoid desoxycorticosterone pivalate (DOCP) to aggravate hypertension and systemic inflammation. Mice developed typical HFpEF features including pulmonary congestion, diastolic dysfunction, LV hypertrophy, fibrosis, elevated levels of natriuretic peptides, and systemic inflammation. Moreover, treatment with β-hydroxybutyrate attenuated NLRP3 inflammasome activation by reducing mitochondrial hyperacetylation and dysfunction (Deng et al., 2021).

Several other multiple-hit models of HFpEF have been described which do not approximate clinical standards of HFpEF to the degree of those described previously, but nevertheless serve as important preclinical models for defining HFpEF pathophysiology. Notable examples include mice given HFD + Ang-II infusion (Reddy et al., 2018; Piek et al., 2019), uninephrectomy + aldosterone infusion (Tanaka et al., 2014; Valero-Munoz et al., 2016), and the deoxycorticosterone acetate salt-sensitive model (Mohammed et al., 2010). The Zucker fatty and spontaneously hypertensive (ZSF1) rat is another commonly employed model that develops HFpEF-like symptoms owing to diabetes and hypertension (Schmederer et al., 2018; Schauer et al., 2020). A recent study by Wu et al. highlighted the role of endothelial sirtuin 6 (SIRT6) impairment in HFpEF development using a diabetic, HFD mouse model in which restoration of endothelial SIRT6 function reduced cardiac lipid accumulation and improved cardiac function, thereby directly linking diabetic ECD to HFpEF-like symptoms (Wu et al., 2022).

Despite their closer approximation of human physiology, animal models are resource-intensive and limited by known species differences. Recent developments in three-dimensional culture systems and high-throughput screening platforms using patient-derived induced pluripotent stem cells (hiPSC) are making *in vitro* models increasingly feasible as complementary to animal-based models.

### 5.3 *In Vitro* models of EC-CM signaling and cardiac disease

Developing translatable models of EC-CM interactions *in vitro* requires careful consideration of the complexity of the myocardial tissue niche. The possibility of including multiple cellular populations, external signaling factors, and/or dynamic mechanical forces has only recently become feasible. Furthermore, the opportunity to develop sophisticated myocardial model systems composed exclusively of human cell lines provides a highly relevant platform for exploratory therapeutic studies and disease modeling. Indeed, the use of data from hiPSC-CM in regulatory submissions to the U.S. Food and Drug Administration (FDA) has steadily increased in recent years and represents an important corollary to other nonclinical data (Yang X. et al., 2022).

Two-dimensional (2D) cultures of both primary CM and hiPSC-CM have been used extensively in electrophysiological (Morton et al., 2014) and cardiotoxic drug screening (Gintant et al., 2017; 2019). For assessment of the influence of EC-CM interactions in physiologic alterations and cardiac remodeling, *in vitro* systems require inclusion of (at a minimum) CM and EC.

Coculture of CM and EC in a traditional monolayer format has substantiated observations about EC-CM crosstalk that are challenging to clearly define in animal models. For example, EC have been shown to protect cocultured CM from hypoxia-reoxygenation injury via both hypoxia-inducible factor (HIF1α) (Leucker et al., 2011) and NO signaling (Leucker et al., 2013). Similarly, transwell coculture systems have implicated EC-derived paracrine factors in control of CM cytoskeletal organization (Zhang et al., 2015) and natriuretic peptide expression (Jiang et al., 2019).

Moreover, the use of patient-derived iPSCs allows for *in vitro* molecular assessment of genetic diseases. Models of several inherited cardiomyopathies including HCM (Lan et al., 2013), dilated cardiomyopathy (DCM) (Sun et al., 2012; Lee et al., 2019), and arrhythmogenic cardiomyopathy (ACM) (Te Riele et al., 2017) have been established in 2D monoculture using hiPSC-CM. Inclusion of EC in coculture with hiPSC-CM enables the study of EC-CM crosstalk in disease pathogenesis. Transcriptional profiling of hiPSC-derived EC (hiPSC-EC) from patients with mutations in LMNA identified downregulation of KLF2 as a driver of clinical ECD, which was rescued with lovastatin treatment. Additionally, co-culture of lovastatin-treated patient hiPSC-EC and hiPSC-CM restored CM contractile function, indicating a direct role of KLF2-mediated NO dysregulation in the LMNA cardiolaminopathy phenotype (Sayed et al., 2020).

Despite their convenience and cost-efficiency, conventional 2D culture systems are limited in their capacity for modeling cardiac disease states as they cannot adequately recapitulate morphogenetic or physiological tissue processes, nor do they allow for assessment of the complex cell-cell and cell-extracellular matrix (ECM) interactions that take place within the heart. Thus, three-dimensional (3D) cardiac tissue models have emerged in recent years as a potential experimental platform that better resembles the organization of normal cardiac tissue.

Current 3D cardiac culture approaches include aggregation of spheroids and organoids, engineered heart tissues (EHT), microfluidics, bioprinting, and electrospinning, among others (Tenreiro et al., 2021; Mohr et al., 2022). The terms “cardiac spheroid” and “cardiac organoid” are used somewhat interchangeably, however the term spheroid most often refers to aggregation of CM with or without other pre-differentiated cells (e.g., EC, fibroblasts), whereas organoid is typically used in reference to more complex 3D cell structures generated via self-aggregation and differentiation of induced-pluripotent stem cells (iPSCs). Given their relatively low resource requirements and fabrication steps, organoid and spheroid systems have been at the forefront of considerable effort towards developing a human-relevant model of vascularized myocardium. Current state-of-the-art cardiac organoid systems have been developed for applications such as drug screening, cardiogenesis, and disease modeling, all of which have important implications for our understanding of EC-CM interactions (Arslan et al., 2022).

Because of the substantial role of non-myocytes in modulating cardiac contractility and electrical activity, concerns have been raised that CM monoculture alone is insufficiently sensitive for cardiotoxicity assessment (Pointon et al., 2015). Similarly, many chemotherapeutic agents induce cardiotoxicity by disruption of the vascular endothelium and/or cardiac fibroblasts, most notably doxorubicin and other anthracyclines (Luu et al., 2018; Zhang et al., 2019). Heterocellular cardiac organoids including hiPSC-CM, adipose-derived stem cells, EC, and fibroblasts have recently been demonstrated as a next-generation drug screening platform that better captures the composition of the adult heart (Richards et al., 2020). Other notable tri-lineage, vascularized culture systems employed for cardiotoxicity analysis include micropatterned 3D scaffolds (Wanjare et al., 2019) and multilayered tissue aggregations (Tadano et al., 2021).

In recent years, many groups have reported generation of self-organizing organoid systems termed “gastruloids” or “cardioids” involving careful orchestration of developmental cues to achieve formation of primitive chamber-like structures and organized differentiation of epicardial, myocardial, and endocardial layers (Rossi et al., 2021). These systems have already shown immense promise in modeling cardiogenesis (Hofbauer et al., 2021) and congenital defects (Drakhlis et al., 2021). However, these systems are currently intended to capture the earliest stages of the developing heart, rather than mimic mature cardiac tissue.

Patient specific hiPSC-derived organoid systems have also demonstrated great utility in disease modeling. Buono et al. reported generation of trilineage organoids using hiPSC-CM from a patient with HCM, which displayed arrhythmogenicity when compared to organoids generated using healthy donor hiPSC (Filippo Buono et al., 2020). In another study, inclusion of hiPSC-derived cardiac fibroblasts from patients with ACM induced loss of Cx43 and cardiac arrhythmia in tri-lineage organoids containing healthy CM, implicating cardiac fibroblasts in driving the ACM phenotype (Giacomelli et al., 2020). Further development of such disease models will be crucial for correlating long-standing clinical observations with a molecular phenotype, opening the door for high-throughput therapeutic screening and personalized medicine. Similarly, endothelium-specific genetic modifications applied to heterocellular organoid systems will likely help to corroborate preclinical findings in animal models.

As a newly emerging field, several commonly cited limitations currently hinder the physiological relevance of spheroid and organoid-based systems, especially in the context of modeling EC-CM interactions in cardiac pathology. First, most cardiac spheroid and organoid models more closely resemble immature fetal cardiac tissue than adult cells given their hiPSC origin (Van Den Berg et al., 2015). Thus, promoting cellular maturation to a more adult-like phenotype has formed a main thrust of the field. Cardiac organoids incorporating hiPSC-EC demonstrate improved expression of cardiac ion channel and Ca^2+^-handling genes (Giacomelli et al., 2017), whereas additional inclusion of hiPSC-derived cardiac fibroblasts (hiPSC-CF) improved hiPSC-CM sarcomeric alignment, electrical maturation, mitochondrial respiratory capacity, and intracellular cAMP signaling (Giacomelli et al., 2020). Electrical stimulation of engineered cardiac tissues has also been reported to improve contractile maturation, but long-term pacing has not yet been assessed in organoid systems (Shen et al., 2022; Sesena-Rubfiaro et al., 2023). Second, insufficient vascularization of organoids limits their growth and functional potential. While many groups have achieved formation of lumenized or non-lumenized vascular structures within cardiac organoids, a fully perfusable system by means of microfluidics or pumps will permit modulation of fluid shear stresses, circulating factors, and immune cells. To date, very few reports have been made of such a system. A recent model by Arslan et al. involved pre-vascularized, tri-lineage cardiac microtissues (MT) cocultured with vascular cells within a fibrin hydrogel microfluidic and demonstrated perfusable vascular networks in and around the gel-laden cardiac MT. Further, perfusion of L-NAME or IL-1β induced functional changes in the vascularized cardiac MT, showing promising results as a model system of EC-CM paracrine signaling (Arslan et al., 2023). Another similar non-organoid system was developed by King et al. involving aggregation of hiPSC-CM, human cardiac microvascular EC (hCMVEC), and human left ventricular fibroblasts (hLVFB) in a three-dimensional fibrin matrix situated within a microfluidic device. This system developed fully perfusable microvascular networks that were shown to increase hiPSC-CM Ca^2+^ transient amplitude and abbreviate contractile time to peak as compared to nonperfused controls (King et al., 2022). Future standardization of such designs will provide important clues regarding the influence of fluid flow and circulating factors in transmitting signals to the subjacent myocardium. Third, given the substantial inflammatory and immunological components of cardiovascular remodeling, introduction of an immune system is necessary for complete recapitulation of the cellular environment of the myocardium. This principle overlaps with the issue of perfusable vasculature discussed above. To date, no organoid culture systems have studied the inclusion of inflammatory cells, although this paradigm has been repeatedly cited as a major limitation of current organoid models and will likely be explored as perfusable systems are developed further (Zhao et al., 2021; Sahara, 2023).

## 6 Discussion

Given their abundance and close association with cardiomyocytes in the heart, it is no surprise that endothelial cells exert a substantial degree of control over the cardiomyocyte phenotype in embryonic development, physiology, and disease. This control is currently understood to be primarily mediated by paracrine signaling, however there is growing evidence suggesting a role of autocrine mechanisms and direct EC-CM junctional communication. Advances in clinical diagnostics and preclinical research over the last 2 decades have challenged the paradigm of cardiac disease as a primarily myocyte-driven pathological process. Indeed, endothelial dysfunction often preempts overt cardiac symptoms, and has increasingly been appreciated as a strong predictive factor of disease prognosis.

Technologies such as single-cell transcriptomics will be increasingly important in defining the earliest changes within the heterocellular myocardial niche during endothelial dysfunction, tissue remodeling, and hypertrophy (Ren et al., 2020; Koenig et al., 2022). Accordingly, amelioration of endothelial dysfunction in the earliest stages of disease is an enticing prospect for halting further progression. While targeted therapies designed to relieve endothelial activation and dysfunction have thus far yielded discouraging results as treatment for many types of heart disease, these findings are likely due to the complexity and overlapping nature of the dysregulated signaling pathways that characterize endothelial dysfunction, as well as a dearth of sufficient preclinical models.

Conditions such as INOCA/ANOCA, HCM, and HFpEF are understood to be multifactorial diseases which develop as a consequence of several aggravating comorbid states. Importantly, this means that such diseases are challenging to fully recapitulate in animal models without introducing several of these risk factors in parallel. To date, no animal studies have thoroughly investigated ECD in the context of INOCA/ANOCA or HCM, however several promising “multi-hit” systems recapitulating the HFpEF phenotype have been established *in vivo*. The advent of three-dimensional culture systems and hiPSC in recent years has paved the way for sophisticated, scalable culture systems that can be engineered to interrogate specific biological questions focused on the EC-CM interaction. For the purposes of modeling the role of ECD in cardiac disease, key future directions include 1) achieving structural and functional maturation of constituent organoid cell populations; 2) generation of cardiac organoids with a fully perfusable vascular system, given the weighty involvement of systemic and circulating components involved in complex cardiac diseases; and 3) addition of immune cells such as neutrophils, monocytes and T-lymphocytes into an integrated flow system to enable modeling of the downstream cardiac impact of endothelial activation and dysfunction.

In summary, endothelium-derived signaling cues closely regulate cardiac growth, remodeling, and function, the disruption of which is driven by a range of established cardiovascular risk factors. Endothelial dysfunction can be diagnosed and defined clinically but does not yet substantially inform treatment strategies. Moreover, endothelial dysfunction has been repeatedly implicated in complex cardiac diseases such as INOCA/ANOCA, HCM, and HFpEF. Unfortunately, most endothelium-targeted therapeutics have thus far yielded neutral or negative results, likely due to an incomplete picture of the interactions between signaling pathways and the multifactorial nature of these diseases. Recent advances in multi-hit animal models and perfusable *in vitro* culture systems have begun to identify novel therapeutic strategies that will further clarify the potential for effective cardiovascular treatments based around amelioration of endothelial dysfunction.
